# Supplementary material for: Effect of operational parameters, characterization and antibacterial studies of green synthesis of silver nanoparticles using Tithonia diversifolia
Source: PeerJ. 2018 Oct 30;6:e5865. doi: 10.7717/peerj.5865 (PMC6214226; doi:10.7717/peerj.5865)
Supplement: Supplemental Information 1 [file peerj-06-5865-s001.docx]

**SUPPLEMENTARY DOCUMENT**

**S1: Procedure of Experimental Optimization of Various Operational Parameters for the Synthesis**

**Effect of Concentration**

The effect of concentration was investigated by varying the concentration of AgNO_3_ used in the following order 0.001 M, 0.002 M, 0.004 M, 0.006 M, 0.008 M and 0.01 M AgNO_3_, all reacted with the leaf extracts at the ratio 1:9. The effect of concentration was done to determine which concentration will give better yield of Ag nanoparticles.

**Effect of Contact time**

The effect of contact time was done to effectively study the rate at which the Ag nanostructures are formed. This will be done by reacting the extract and AgNO_3_ and then agitating and left to stand, an aliquot was taken after 30 minutes and scanned on the Biochrom Libra PCB 1500 UV-VIS spectrophotometer to determine the wavelength of formation of Ag structures, then after every 60 minutes for the 24 hours monitoring and also another reading will be taken after agitation at 90 minutes and 24 hours UV-Vis measurement was done to determine the formation of structures at this particular time respectively.

**Effect of Volume Ratio**

Effect of volume ratio was done to determine the volume of extract to AgNO_3_ at which the most yield of silver nanoparticles is obtained. This was done by varying the volume of the extract and the AgNO_3_ in the ratio shown in the table below.

Table S1: Volume ratio variations of extract to AgNO_3_ solution.

| Volume of Extract (mL) | 10 | 20 | 30 | 40 | 50 | 60 | 70 | 80 | 90 |
| --- | --- | --- | --- | --- | --- | --- | --- | --- | --- |
| Volume of AgNO_3_ (mL) | 90 | 80 | 70 | 60 | 50 | 40 | 30 | 20 | 10 |

**Effect of Temperature:** The effect of temperature was studied at room temperature, 45 ^o^C and 55 ^o^C. 10 mL of the extract was measured and poured into a clean beaker placed in a water bath and the temperature was regulated to 45 ^o^C and 55 ^o^C the extract was left to attain the temperature of the water bath then 90 mL of 0.001 M AgNO_3_ was added with swirling. After 10 minutes of reacting the wave scan was done using a Biochrom Libra PCB 1500 UV-VIS spectrophotometer to monitor the growth of silver nanostructures.

**S2: Anti-Microbial Studies**

The antimicrobial studies of *Tithonia diversifolia* synthesized silver nanoparticles (TD-AgNPs) was carried out by testing TD-AgNPs against multi drug resistant microorganisms (MDRM) which include *Escherichia coli, Salmonella typhi, Salmonella enterica* and *Bacillus* using the agar well diffusion method as reported by (Oluwaniyi *et al.,* 2015)

**S3: Preparation of Nutrient Agar**

LAB M (UK) nutrient agar (LAB008) was prepared according to manufacturer’s direction. 75% ethanol was prepared and used to swab the work desk to sterilize the work area. 2.8g was weighed and poured in a clean Erlenmeyer flask then dissolved in 100 mL of distilled water and swirled a little. Then opening of the flask was tightly corked with cotton wool and heated for few minutes to dissolve the solid particles to give a clear solution before placing in an autoclave and sterilized for 15 minutes at 121 ^o^C and cooled for 30 minutes then poured in plastic petri dishes and left to from gel.

After the agar solidified, the microorganisms were carefully spread using a wire loop on the surface of the agar using a technique called “Streaking” and then carefully placed in the incubator to be incubated for 24 hours at 37 ^o^C.

**S4: Preparation of nutrient broth**

Lab M nutrient broth ‘E” (LAB 068) was used for the sub-culturing of the microorganisms, 1.3 g of the nutrient broth was weighed in an aluminium foil sheet and transferred into a conical flask, 100 mL of distilled water was added to the conical flask and swirl a little, sterile cotton wool was used to tightly cork the opening of the conical flask. The solution was sterilized in an autoclave for 15 minutes at 121 ^o^C and then left to cool to suitable temperature for the introduction of the microorganisms. 5 mL of the nutrient broth was measured and poured into 4 test tubes and labelled accordingly.

The microorganisms were carefully introduced into the broth from the cultured plates with a wire loop by scraping the surface of the agar lightly and inserted into the test tubes with the corresponding microorganism labelled on the plates. The wire loop was constantly flamed to red hot to avoid introducing different organisms in one test tube, the four test tubes were tightly corked with cotton wool after the whole process and incubated in an incubator at 37 ^o^C for 24 hours. A cloudy like solution in the test tubes indicated the presence of the microorganisms in the broth.

**S5: Preparation of Mueller-Hinton agar**

3.8 g of LAB M Mueller Hinton agar (LAB 039) was weighed on an aluminium foil and poured into a clean conical flask, 100 mL of distilled water was measured and added to the conical flask. The solution was swirled a little and sterilized in an autoclave for 15 minutes at 121 ^o^C. It was then stored in a refrigerator for further use.

**S6: Preparation of TD-AgNPs and Positive control agent (**Ciproflaxcin)

The synthesized nanoparticles were air dried for 24 hours in an evaporating dish, 0.2 g of the dried nanoparticles was weighed and diluted with 10 mL of deionized water in a 100 mL beaker. For the preparation of the positive control, 1 tablet of ciproflaxcin was crushed using mortar and pestle, 0.2 g of the crushed Ciproflaxcin tablet was weighed and transferred into a clean 100 mL beaker.

**S7: The Anti-microbial Studies**

The antimicrobial activity was carried out using agar well diffusion method. The Mueller Hinton agar was reheated in the autoclave to melt the solid agar then it was left to cool to a suitable temperature, the test tubes containing the cultured microorganisms were removed from the incubator and spun on a shaker to evenly mix the content, 0.2 mL and 1 mL of each microorganisms was taken and placed in two clean plastic petri dishes and labeled accordingly, then Mueller Hinton agar was added to the plates and the mixture was swirled clockwise, anticlockwise and side ways to ensure a well spread effective mixture of the microorganism in the agar. It was left to solidify. After solidifying 6 holes were bored in the agar using a sterile cork borer of 0.2 mm, 0.2 mL of the TD-AgNPs solution, TD leaf extracts, the positive control (ciproflaxcin) and negative control (sterile water) were introduced into the wells and labeled accordingly. The plates were left to diffuse for 1 hour before placing them in an incubator at 37^o^C for 24 hours. After the incubation period, the mean diameters of the zones of inhibition around the wells were recorded, the values were recorded.

**S7: Supplementary Result**

The antimicrobial studies of TD-AgNPs against gram positive and gram negative micro-organisms was carried out. Shown in Table 1 is the zone of inhibition of antimicrobial activity of TD-AgNPs together with the positive control while TD extract and sterile water (negative control) showed no zero activity.

Table S2: Growth inhibition (Antimicrobial activity) of samples against multidrug resistant microorganisms

| Samples | Zone of Inhibition (mm) | | | |
| --- | --- | --- | --- | --- |
|  | E.coli | S.typhi | S.enterica | Bacillus |
| TD extract | 0 | 0 | 0 | 0 |
| TD-AgNPs | 12 | 11 | 13 | 15 |
| Positive control  (Ciproflaxcin) | 30 | 27 | 23 | 33 |
| Negative control  (Sterile water) | 0 | 0 | 0 | 0 |


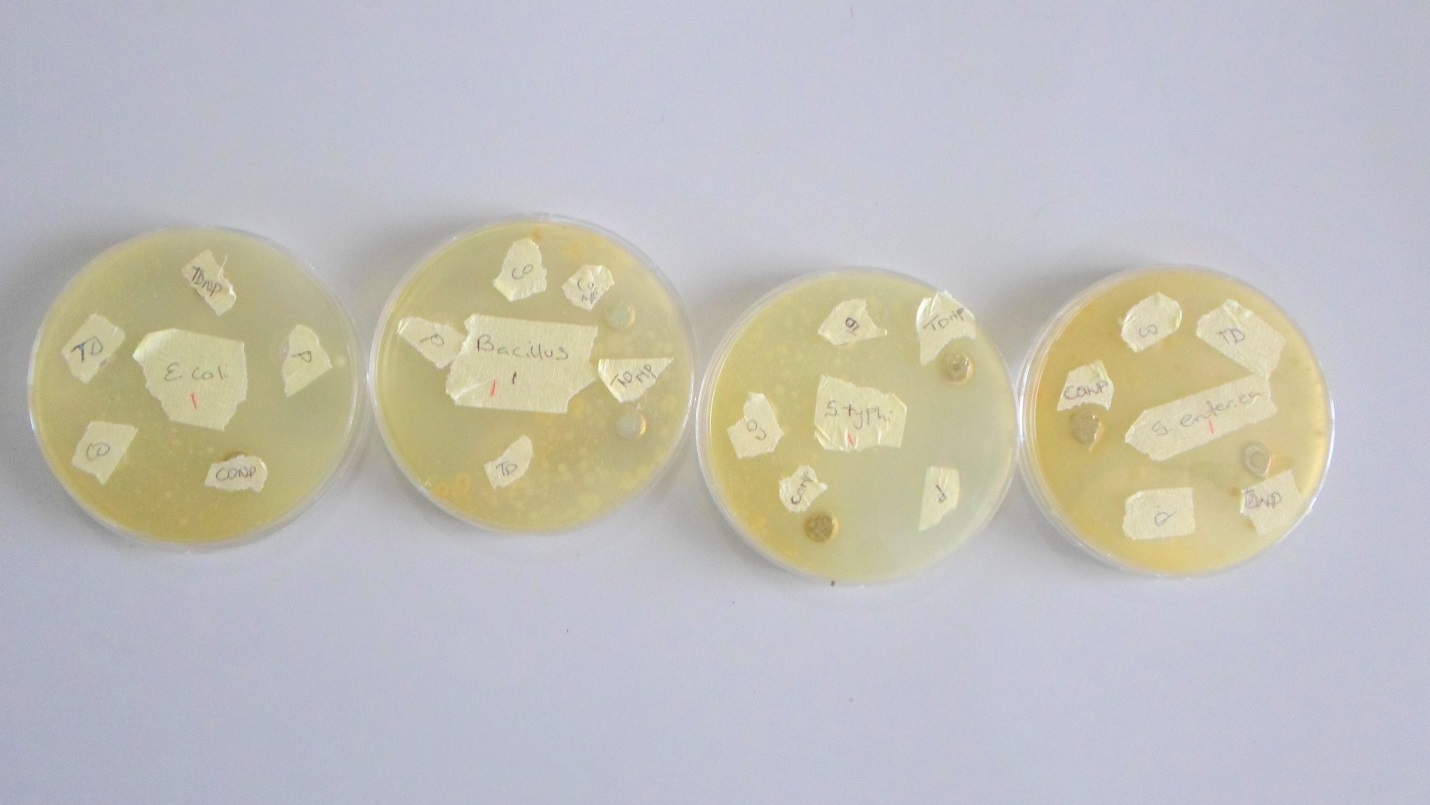


Figure S1: The plates showing the various zones of inhibitions for different bacteria. From left *E.coli, Bacillus, Salmonella typhi and Salmonella enterica.*

**S8: Raw Results of the Characterization:**

Presented in Fig. S2 – S7 are the raw results of the characterization discussed in the manuscript

Figure S2: UV-Vis Spectra of TD-AgNPs with SPR peak at 435 nm.


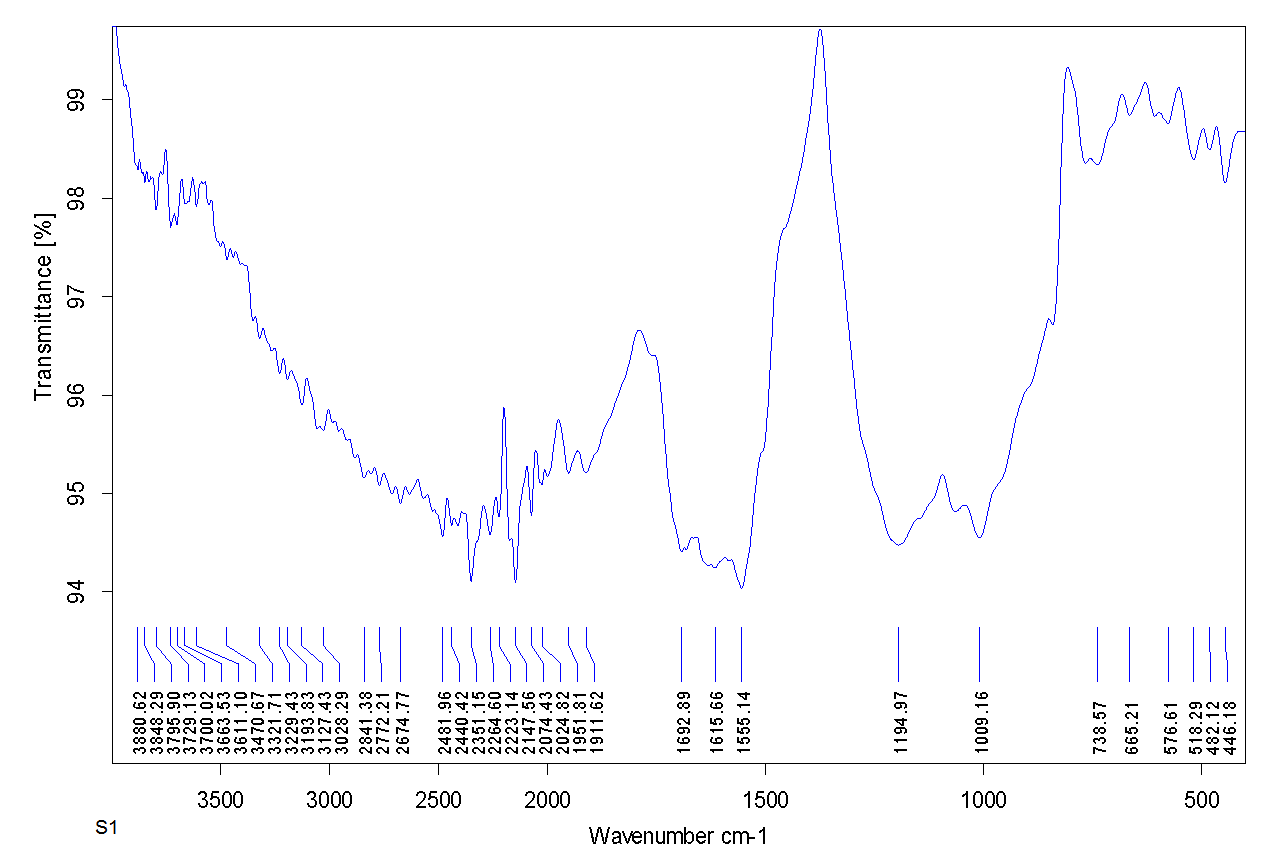


Figure S3: FTIR spectrum of TD-AgNPs

**
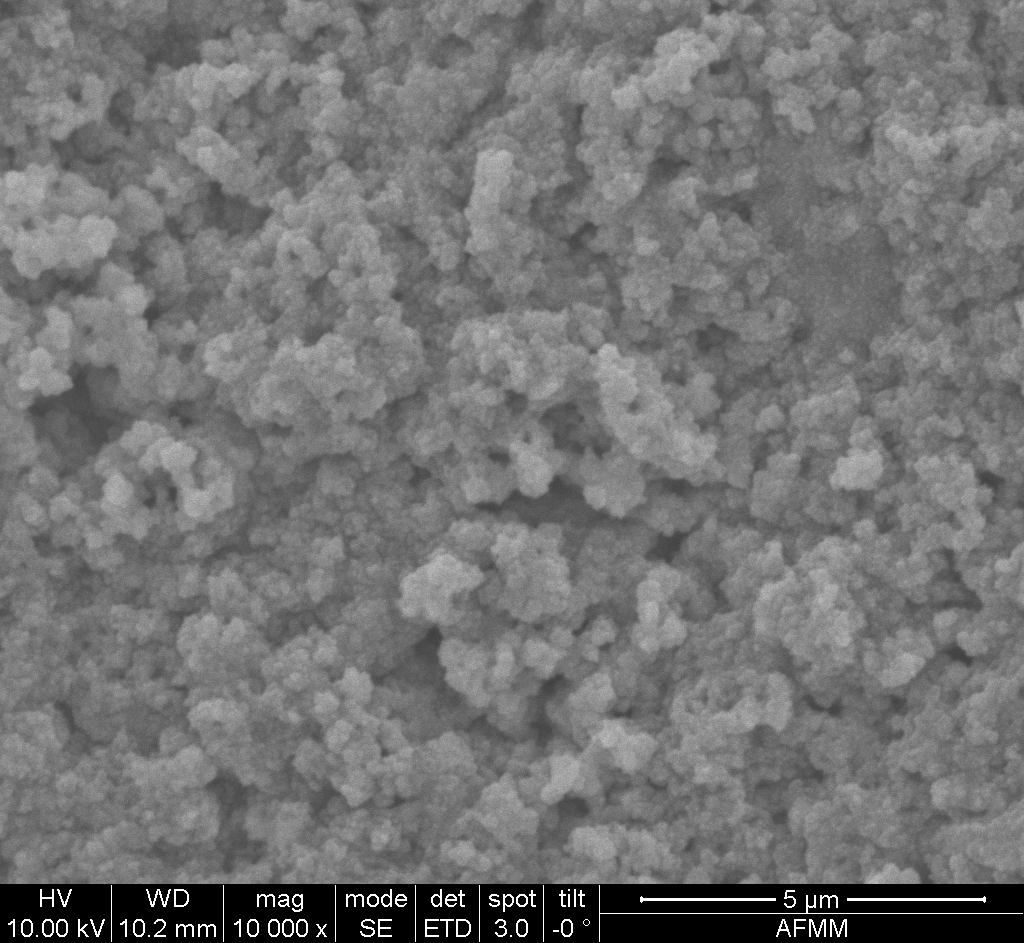
**

Figure S4**:** SEM image showing the surface morphology of TD-AgNPs

Figure S5**:** EDX spectrum showing the dominance of Ag in TD-AgNPs.





Figure S6 XRD spectra of TD-AgNPs


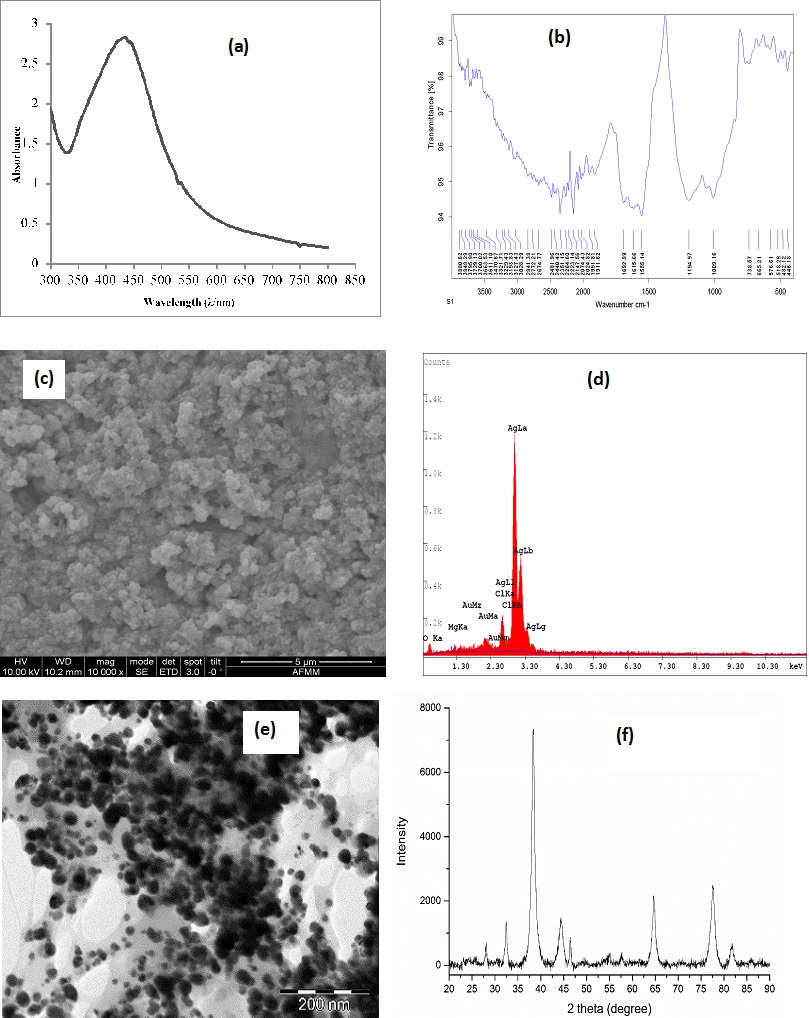


Figure S7: TEM image of AgNPs

S9: Results of the operational parameters relevant to the synthesis of TD-AgNPs.

**(B)**

**(A)**

**S9.0 Phytochemical Screening**

Phytochemical screening was done to identify the presence of phenols, saponins, triterpenes, flavonoids, alkaloids and steroids in both plants leaf extracts. Preparation for the test was done by pouring 3mL of the leaf extracts into separate test tubes and diluting with 2-4 mL deionized water. The various test carried out following the procedures listed below:

**S9.1 Test for Tannins and Phenolic compounds**

Few drops of 3% FeCl_3_ were added to 1mL solution of the extract and agitated a little. The appearance of a deep blue coloration shows the presence of tannins and Phenolic compounds.

**S9.2 Test for Saponins**

Froth’s test was done on both leaf extracts; a portion of the solution of both extracts was put in two clean test tubes and was shaken vigorously then left for few minutes. The presence of a stable froth confirms the presence of saponins.

**S9.3 Test for Triterpenes**

1 mL of chloroform was added to 1mL of the solution of extract then reacted with 1mL of conc. H_2_SO_4_ which was carefully poured into the test tubes by carefully sliding it down the walls of the test tube in the solution. The appearance of red colour indicates the presence of triterpenes.

**S9.4 Test for Flavonoids**

**With lead acetate**: Lead acetate solution was added to 1ml of the solution of the extracts. The formation of a yellow precipitate confirms the presence of flavonoids.

**With NaOH**: Few drops of NaOH were added to the solution of the extract with the lead acetate to decolorize the yellow color formed, a decoloration confirms the presence of flavonoids.

**S9.5 Test for Alkaloids**

Dilute HCl was added to the solution of the extract, shaken and filtered. The filtrate was kept and the Meyer’s test was done on it by measuring 2mL of the filtrate and adding Meyer’s reagent. The formation of yellow precipitate or turbidity indicates the presence of alkaloids (Sofowora, 1993)

**S9.6 Test for Steroids**

Libermann-Buchard test was done to determine the presence of sterols and steroids by mixing 2mL of the solution of the extract with 1ml acetic anhydride and heating for few minutes then cooled, Concentrated H_2_SO_4_ was added in few drops by sliding down the side of the test tube. The appearance of a blue color indicates the presence of steroids.
