# Supplementary figures and images for: Effect of operational parameters, characterization and antibacterial studies of green synthesis of silver nanoparticles using Tithonia diversifolia
Source: PeerJ. 2018 Oct 30;6:e5865. doi: 10.7717/peerj.5865 (PMC6214226; doi:10.7717/peerj.5865)

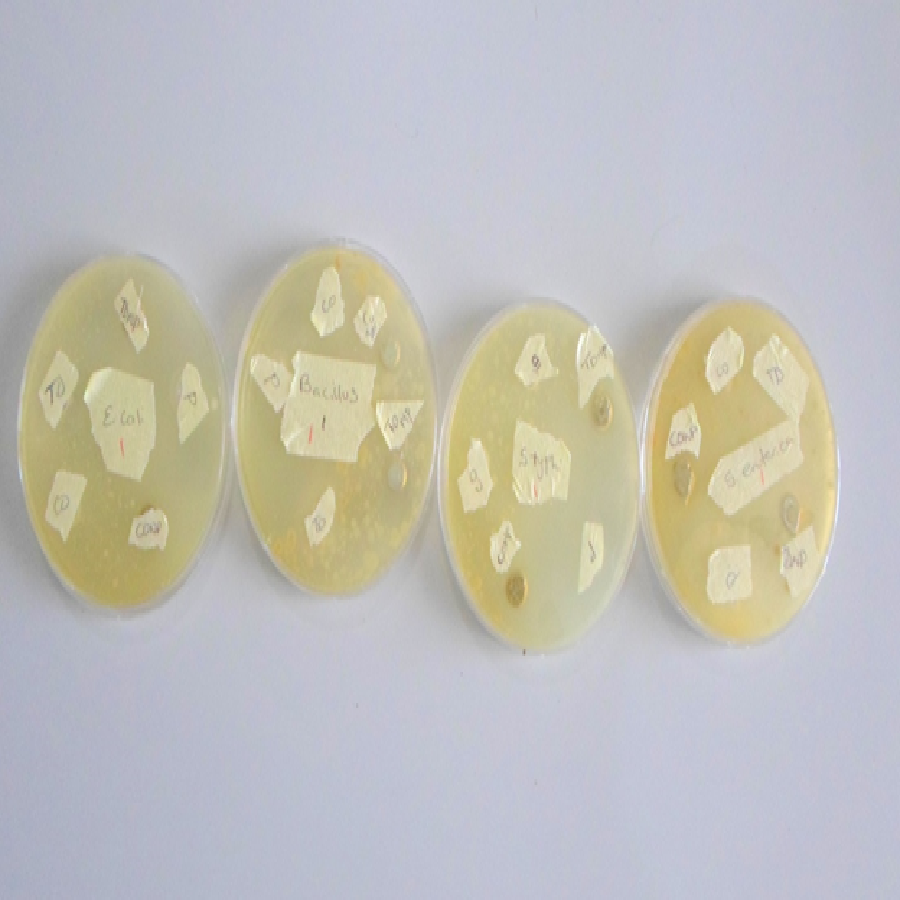

Supplement: Supplemental Information 2 [file peerj-06-5865-s002.png]
